# From aspiration to implementation of the 30% solution

**DOI:** 10.1093/nsr/nwad195

**Published:** 2023-07-17

**Authors:** Stuart L Pimm

**Affiliations:** Nicholas School of the Environment, Duke University, USA

In closing the COP15 of the Convention on Biological Diversity in late 2022, Huang Runqiu, China's Minister of Ecology and Environment and COP15 president, celebrated the agreement to protect ≥30% of the world by 2030. Recognizing Indigenous and traditional territories, where applicable, he said the agreement marked a ‘historic moment’ in global efforts to save nature, calling the deal ‘a package we can all be proud of’ [1]. The challenges in implementing this solution are formidable, as a recent international effort led by Xiaoli Shen, Mingzhang Liu and Keping Ma explains [2]. This timely article addresses the critical issue of how to disaggregate the global 30% target at the country level and identifies the different responsibilities of each country and the possible paths for implementation.

Countries differ enormously in size. There are small island nations such as Singapore and oceanic islands, many <1000 km^2^. At the other end are seven countries—Russia, Canada, China, the USA, Brazil, Australia and India—comprising almost half Earth's ice-free land surface. The island states may be tiny in comparison but their 200-nautical-mile economic exclusion zones can protect a lot of ocean. Protecting 30% of the world's oceans is also a COP15 aspiration.

Countries also differ in the size of their economies. The richest—the USA, Australia and various European nations—have a per-capita Gross National Income of >$45 000. Many African countries have less than a tenth of that, and Madagascar, Mozambique and the Democratic Republic of Congo have <$1500.

Shen *et al.* sensibly also look at vulnerable carbon stores—concentrated in the Amazon, Congo and Southeast Asia and the boreal forests of Canada and Russia. About two-thirds of all terrestrial species live in tropical moist forests. Their destruction causes species extinctions and results in ∼10% of the global carbon dioxide emissions. That heats the planet causing more extinctions as species ranges shrink. The disproportionate heating of the Arctic, where vast carbon deposits in the soils may oxidize, diminishes ice cover and further increases warming.

Finally, Shen *et al.* consider the complexities of these factors and their impact on biodiversity. Tropical countries, especially forested ones, dominate the league tables of species, but numbers alone do not tell the full story. Special places, called biodiversity hotspots, hold large numbers of species that occur nowhere else [3]. They include small island nations, but more familiar are places such as Madagascar, the countries of the northern Andes, West Africa and insular Southeast Asia.

Shen *et al.* argue that there must be global, cooperative actions to achieve global conservation. Some of the most important countries for biodiversity have small economies and need help from more affluent nations. There are vital synergies in protecting species and sequestering carbon. Species-rich countries often have large stores of carbon in their forests, the protection of which is essential to stemming global climate disruption.

There has been much progress in the decades before COP15, resulting in the protection of 17% of the land surface. But looking at which areas are protected shows that they are predominantly cold or dry places: forests are relatively poorly protected. China has made much recent progress in protecting forests, especially those with the species at greatest risk of extinction [4]. It, and all other nations, will need to continue to be strategic in how they implement the 30% if it is to achieve the COP15’s goal of stopping extinctions. Area alone will not be enough.
